# Simple bone cyst: description of 60 cases seen at a Brazilian School of Dentistry and review of international literature

**DOI:** 10.4317/medoral.23638

**Published:** 2020-07-19

**Authors:** Lívia Bonjardim Lima, Silas Antonio Juvencio de Freitas Filho, Luiz Fernando Barbosa de Paulo, João Paulo Silva Servato, Roberta Rezende Rosa, Paulo Rogério de Faria, Adriano Mota Loyola, Sérgio Vitorino Cardoso

**Affiliations:** 1 Oral Pathology Area, School of Dentistry, Federal University of Uberlândia, MG, Brazil; 2 Bio-pathology Area, School of Dentistry, University of Uberaba (UNIUBE), MG, Brazil; 3 Department of Morphology, Biomedical Science Institute, Federal University of Uberlândia, MG, Brazil

## Abstract

**Background:**

The aim of this study was to describe the relative frequency and the main demographic and clinic-radiographic features related to patients diagnosed with Simple bone cyst (SBC) in an Oral Diagnosis Service in Southeast Brazil and present a review and discussion of international literature on this topic.

**Material and Methods:**

SBC cases from our service encompassing the period between 1978 and 2017 were selected. In addition, a literature search was performed in the Pubmed/MEDLINE online electronic database published between 1951 and 2019.

**Results:**

A total of 2,459 cystic lesions were documented in our service, thus 60 patients were diagnosed with the SBC representing 2.4% of all jaw cystic. Most of cases were asymptomatic. Multiple SBC lesions were seen in two patients (3.4%) and association with cemento-osseous dysplasia was seen in one female patient (1.7%). A total of 793 cases were enrolled in this literature review.

**Conclusions:**

The SBC is an asymptomatic lesion often discovered in routine image exams in young patients. The unilocular, well defined margin with scalloped appearance is characteristic and helps the definition of diagnosis. This review suggests a different epidemiologic trend concerning to the sex and it confirms the posterior region of mandible as the more frequent location. The conservative treatment with limited exploration and curettage remains as the gold-standard treatment.

** Key words:**Simple bone cyst, idiopathic bone cavity, traumatic bone cyst, conservative treatment.

## Introduction

Simple bone cyst (SBC) is defined as an intraosseous cavity of unknown etiology, that is devoid of an epithelial lining, and is either empty or filled with fluid (1,2). Many names have been given to this lesion, such as: solitary (2), hemorrhagic (3,4), unicameral bone cyst (5), idiopathic bone cavity (6) and traumatic bone cyst (7). The last one has been frequently used, but a trauma does not seem to play a role in its pathogenesis (6,8,9).

In the facial skeleton, SBC is most commonly observed in body mandible, during the second/third decade of life, with no gender predilection (1). It is often asymptomatic, with most cases discovered incidentally on radiographs taken for dental purposes (1,3,10,11).

The typical radiographic appearance of SBC is a radiolucent and unilocular lesion expanding between the roots of teeth (1,12), with no or only slight cortical expansion (1,11). Root resorption or displacement are rare (1,13). At surgical exploration, SBC is usually a cavity with altered cortical, empty or filled with variable amounts of serous or sanguineous fluid (1,3,13). This finding is considered a pathognomonic feature for some authors (10,14). Although this, histopathological examination is useful to exclude other diseases (13,15).

Few well documented series of SBC patients are available in the international literature (14,16-18). The aim of this study was to describe the relative frequency and the main demographic and clinic-radiographic features related to 60 patients diagnosed with SBC in the Oral Diagnosis Service of Federal University of Uberlandia, MG/Brazil. The international literature on this topic is also reviewed and discussed.

## Material and Methods

A retrospective descriptive study was developed based on the records from our service, encompassing the period between 1978 and 2017. This investigation was previously approved by local Institutional Reviewer Board for ethics on research (Protocol number: 305/07). All cases originally reported as SBC were retrieved and revised according to the fourth World Health Organization (WHO) Classification of Head and Neck Tumours. Data concerning gender, age, skin color, number and anatomic location of the lesion, history of trauma, symptomatology, association with other bone diseases, radiographic characteristics, treatment, and outcomes were recovered from medical and dental files, histopathological exam requests, and available radiographic exams. Regarding anatomic distribution, each lesion was classified according to its epicentre into anterior, premolar or posterior regions of the maxilla or mandible, as well as the angle or ramus of the mandible. For patients with a concise follow-up, treatment results were evaluated according to the radiographic criteria of Capanna *et al*. (19) (1- Complete healing; 2- Healing with residual radiolucency; 3- Recurrence and 4- No response to treatment).

A literature search was performed in the Pubmed/MEDLINE online electronic database, looking for case series (n = 5 cases or higher) published between 1951 and October 2019 with the terms “simple bone cyst”, “traumatic bone cyst”, “idiopathic bone cavity”, “hemorrhagic bone cyst”, or “unicameral bone cyst” combined with “mandible”, “mandibular”, “jaw” or “maxillofacial”. No restriction of language was made. Case series associated with literature review had only their own sample data collected. Two articles were excluded because a third one (20) written by the same group of authors presented more comprehensive data. For each study, it was collected the data about: Author and year, country, interval of data collection, number of cases, sex and age of affected patients, bone affected, presence of signs and symptoms, vitality of the related teeth, history of trauma and main radiographic features.

## Results

Sixty SBCs cases were found in this Oral Diagnosis Service. In the period mentioned above, 2459 cystic lesions were documented in our service, thus the SBC reported in this case series represent 2.4% of all jaw cystic diagnosed. Main clinical data is presented by Table 1. Most cases were asymptomatic (49/60, 81.7%). In the symptomatic cases, pain or swelling was reported by 10 patients (16.6%); only one person complained about both symptoms together (1.7%).

Multiple SBC lesions were seen in two patients (3.4%) and association with cemento-osseous dysplasia (COD) was seen in one female patient (1.7%).

**Figure 1:**
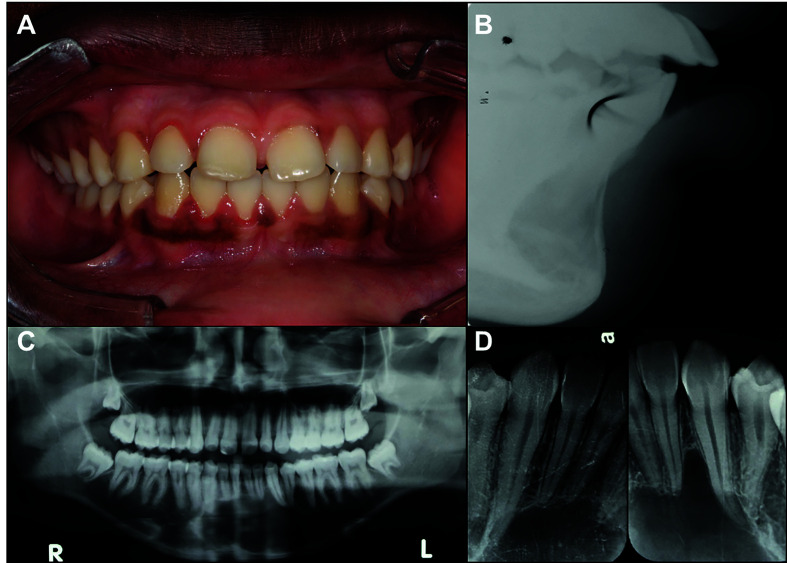
A: Intra-oral aspect of anterior SBC; B: Sagittal Radiographic aspect of the SBC. C: Panoramic radiograph showing SBC lesion in anterior portion of mandible; D: Periapical radiograph presenting scalloped margins between dental elements.

**Figure 2:**
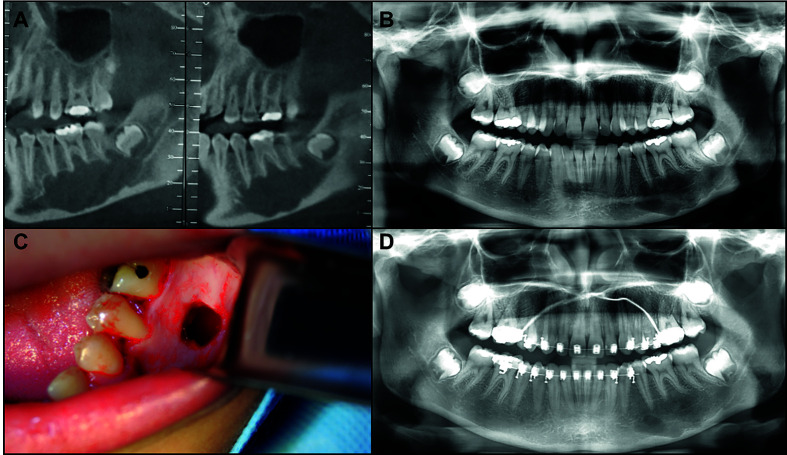
A: Parasagittal tomographic aspect of a mandibular SBC; B: Panoramic radiograph showing SBC lesion in left posterior portion of mandible involving second premolar, first and second molar. C: Surgical exploration of the lesion; D: Pos surgical radiograph evidencing bone formation.

**Figure 3:**
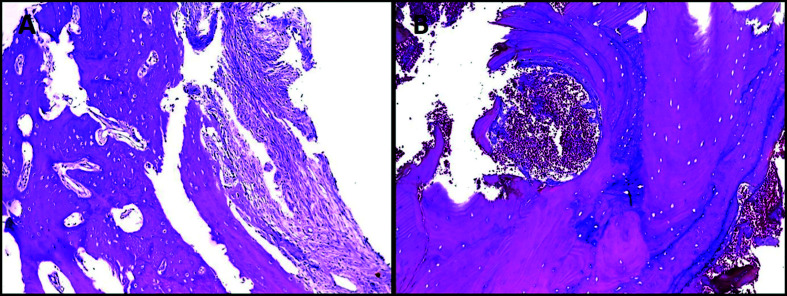
Histopathological features of the SBC. A: Areas of fibrovascular connective tissue with the presence of vital reactive bone trabeculae (Hematoxylin & Eosin stain, ×100); B: Vital bone tissue and hemorrhagic foci (Hematoxylin & Eosin stain, ×100).

Radiographies from 51 cases were available for review, and their information are presented by Table 1. Information about surgical treatment, outcome and follow-up was available for fourteen patients and is presented in Table 2. The clinical, radiographic and treatment aspects of two patients are shown in Fig. 1 and Fig. 2. The Fig. 3 shows the main histopathological aspects of the SBC.

The literature review enrolled twenty-nine articles (1,6-9,14-18,20-38). The clinical and radiographic features of SBC retrieved from the literature review are presented on Table 3 and Table 4, respectively. A total of 793 cases were enrolled in this literature review.

**Table 1:**
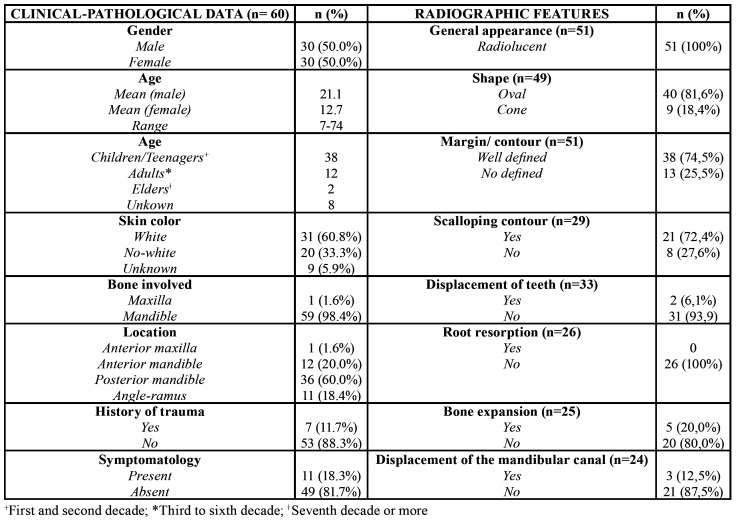
Clinical-pathological data and radiographic features of patients with SBC.

**Table 2:**
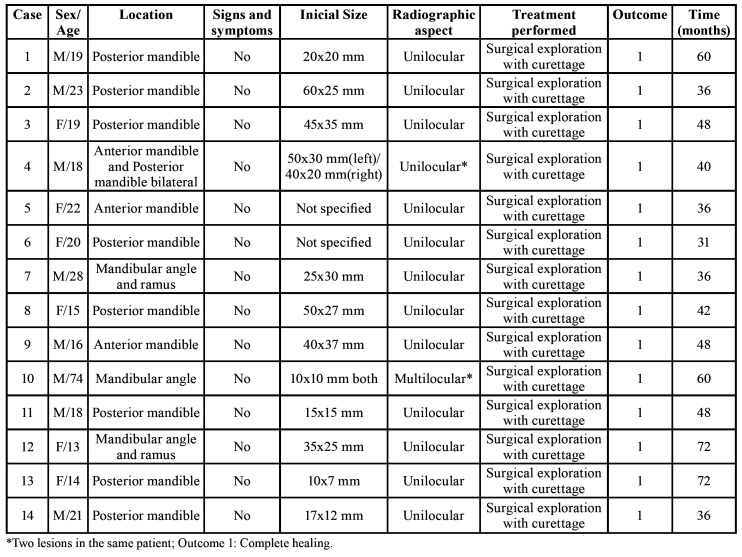
Results of treatments performed utilizing the Criteria of Capanna *et al*.

**Table 3:**
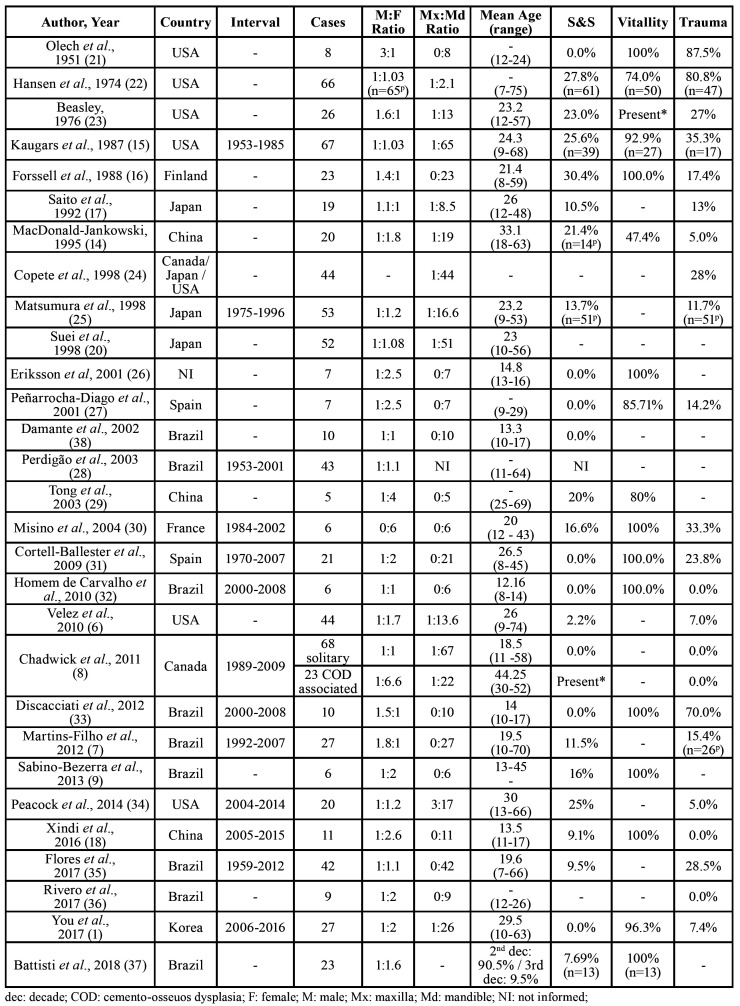
Clinical-pathological data of published series of SBC (1951 to October 2019).

**Table 4:**
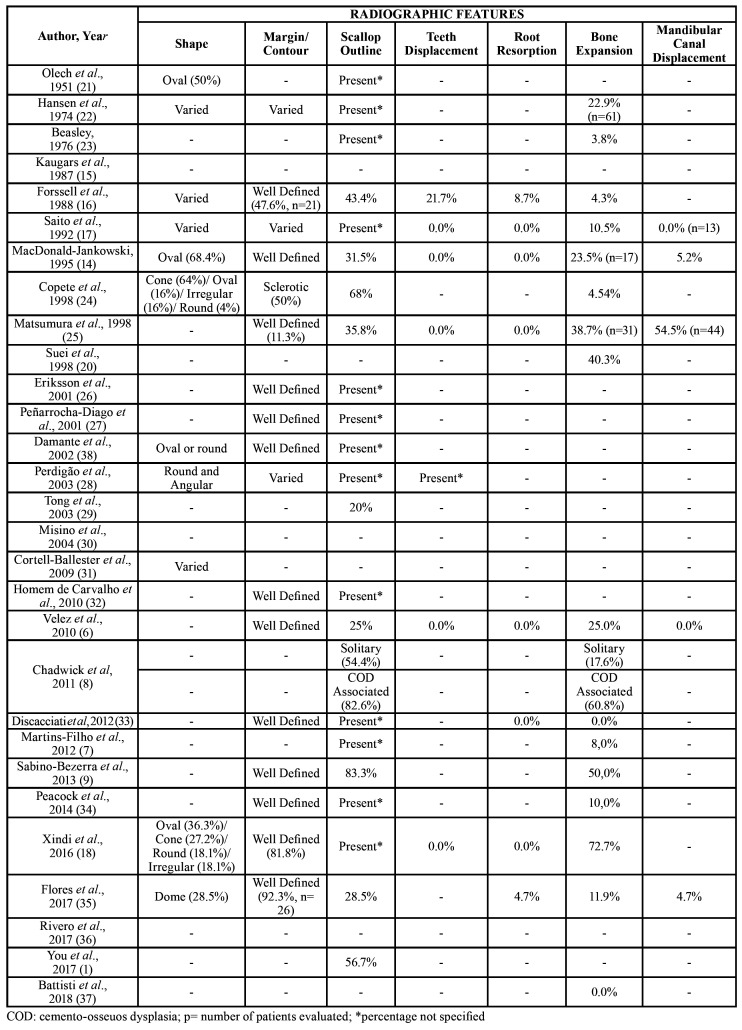
Radiographic features SBC retrieved from literature review (1951 to October 2019).

## Discussion

SBC are uncommon lesions of maxillofacial region. They represented less than 14% of the bone lesions reported by a study conducted for a 15-year period in a Brazilian clinic (7) and 1.48% in a sample of 1283 jaw cysts (17). Similarly, other authors (2,38) reported that SBC comprise only about 1% of all jaw- cysts. Our sample is slightly more representative, with 2.4% of the jaw cysts of the service.

In the past, the most widely accepted etiology hypothesis for SBC was related to traumatism without bone fracture in young patients, which could trigger an intramedullary hemorrhage with hematoma formation and finally development of cavity (2,4). In the present review, seventeen studies reported history of trauma in their casuistry (1,6,7,14-17,21-25,27,31,33). Only three (21,22,33) of them showed indices of more than 50% of trauma history related to their sample. The present series revealed trauma as an etiological factor for only seven cases (11.7%); from these five patients reported that the trauma had occurred in fourth and fifth decade of life (8,4%). The presented findings were against the trauma theory, which states that the trauma must affect only younger people (22). Moreover, the SBC incidence in patients with a history of trauma seems to be the same as that in the general population (1,6,7,14-17,23-25,27,31,34). These inconsistencies show that the role of trauma in the etiopathogenesis of SBC still needs further studies, possibly with a larger number of patients.

Moreover, other authors proposed that the trauma origin is not the most appropriate hypothesis to explain the SBC etiopathogenesis, since their cases were not related to trauma situations (9), while others authors (8) hypothesized that the formation of an empty cavity in bone may be related to the inability of osteoblasts to keep up with the demand for bone mineral deposition during normal remodeling processes, because of constant changes during the development process in young patients and possible an inadequate number of osteoblasts, especially in middle age woman.

The majority of published series are from South America samples (7,9,28,32,33,35-38), followed by Asia (1,8,14,17,18,20,25,29), North-America (6,8,15,21-23) and Europe (16,27,30,31). The present casuistry represents the fourth biggest sample described in the international literature (8,15,22). Most studies (1,6,8,9,14,15,18,20,22,25) show predilection for female patient. In other hand, few studies (7,16,17,21,23,33) reported that SBC are most frequent in male patients. In our sample, there was no male: female predilections, similarly as the epidemiologic characteristics reported by others studies (8,32,38).

About age predilections, SBC was more frequently diagnosed in young adults - in the second and third decade of life (6-8,15-18,20,21,23,25-27,30), but it may affect children and elders with a wide range age distribution. In some works (14,29), a high incidence of SBC in elderly patients were found, especially when it was associated with COD. In our sample the most affected age group was children and teenagers (first and second decade of life), with 38 patients, representing 63.3% of the sample. Concerning to skin color, our study demonstrated a predilection of SBC for white patients. This is in accordance with others studies that also reported this racial tendency (6,15,21,23,33,35).

Most of the retrieved paper, describes that SBC was most commonly found in mandible (1,6-9,14-18,20-27,29-36,38). Only two articles (28,37) did not bring that information. In the present case series, the most affected mandible regions were premolar and molar area, followed by anterior portion, and angle-ramus area. This finding agrees with the characteristics mentioned in the most of reviewed studies (1,6,7,9,14-16,23-25,30-32). Among the evaluated studies, only five (18,22,26,27,33) reported a predilection for the anterior portion of the mandible, with a percentage of at least 60% in their casuistry.

Usually SBC patients, do not develop symptoms or evident clinical signs and the diagnosis is made often by routine radiographic examination (1,3,10,38). Nevertheless, the most of the reviewed studies (1,6-9,17,18,21,25-27,29-33) showed between 0 a 20% of patients with some sign or symptom related to this lesion. The presence the symptomatology ranged from 2.27% to 30.43% in the literature reviewed. In the current study, less than 20.0% of all patients reported at least one symptom (swelling or pain) and only one patient complained of both.

Usually, the radiographic findings of SBC display unilocular radiolucent lesion with well-defined borders and scalloping contour among roots of involved teeth by lesion (1,4). Fourteen articles reported well-defined margin in their sample (6,9,14,16,18,24-27,32-35,38), with a range of 11,32% to 92,30% of occurrence. Scalloped outlining was also frequently mentioned in the retrieved studies (1,6-9,14,16-18,21-29,32-35). These findings also were reported and prevailed in our cases series.

The presence of expansion of cortical margin is variable and can be analyzed either by physical or imaginological examination. The literature review presented a range 3.84% a 72.7% of bone expansion associated with SBC (6-9,14,16-18,20,22-25,34,35) and in this study it was seen in about 20,0% of cases. Some SBC cases can cause inferior alveolar nerve canal dislocations; our literature review reveal inconsistent range of this RX-findings varying from 0 (6,17) and reaching up to 54,5% (25). Our study showed this dislocation in only three patients (5%).

Displacement of teeth caused by SBC was not a common radiographic finding in our series, as well in the majority of reviewed studies, with only two articles (16,28) reporting this event. The vitality of involved or adjacent tooth remained positive in the majority of the cases (1,9,15-18,21,22,26,27,29-33) as well as in the present case series. Although, there was one article (14), which reported vitality rate of only 47.4% of their sample. In our series, even after the surgical treatment, the tooth vitality was maintained, as reported by some articles (16,32). In the same way, a group of authors (22) highlights that the lack of vitality of teeth is not associated with the etiology or pathogenesis of the cyst. Other aspect less commonly described is root resorption. In our sample, there is no patient with this radiographic sign, as reported by six other studies (6,14,17,18,25,33). However, in two studies (16,35) it was described in 8.69% and 4.76%, respectively.

Patients committed with multiple SBC lesions have also been reported by several authors in the literature (1,7,14,17,23-25,28,29,33). In our sample only two patients (3.4%) showed multiple SBC lesions. Multiple lesions have been described in a mild range frequency, varying from 2.2% to 40% of all SBC cases (1,7,14,17,23-25,28,29,33). Four articles (7,23,24,33) described only one case of multiple lesions among their patients, four others studies (1,25,28,29) reported two patients each one and two other articles (14,17) showed three cases of multiple lesions in their series.

The SBC can also occur in synchronism with other lesions, mainly associated with COD (6,8,17,24,29,34). Among a sample of 44 patients, reported in a study (17), a female patient, between the fourth and fifth decade of life, presented multiple lesions of SBC and association with florid cemento-osseous dysplasia (FCOD). Another study showed approximately 14% of the association with FCOD (6). All the lesions were in female patients and among them, 83.33% were african-american (6). A case series from China (29) reported 60% of their patients with multiple lesions, all associated with FCOD. These patients were all female above the fifth decade of life, which is not a common age for solitary SBC (29). Two other articles (8,34) have shown above 20% of association with COD. One of them (34) reported that the association occurred in older african-american patients. In the present study only one patient had multiple lesions. The patient was edentulous and the lesions were localized in posterior region of mandible right and left side. Another patient showed a focal association between SBC and COD. In the present sample, COD was associated with SBC in a 28-years-old female patient, representing 1.7% of the total SBC reported in our service. The variation on the epidemiologic features (age, race, multiplicity) of these patients opens the question about whether these lesions were actually SBC or they could be an early finding of the COD.

In all cases submitted to curettage (Table 2), histopathological analysis revealed areas of fibrovascular connective tissue, sometimes with myxomatous change and often with immature lace-like osteoid or spiky collagen deposits (1,6,7,14-17,21-25,27,31,33). The presence of vital reactive bone trabeculae, and hemorrhagic foci could also be noted (1,6,7,14-17,21-25,27,31,33). In this way, the term "simple bone cyst" is in fact wrong, because the lesion does not have any epithelial lining, and therefore will be better characterized as a pseudo-cystic lesion (1,6,7,14-17,21-25,27,31,33). Interestingly, a greater thickness of fibrous connective tissue present in the bone cavity of these lesions has been associated with older patients and it has been suggested that this microscopic characteristic has an important role in the repair process (28).

The treatment of choice for theses lesions consists in conservative surgical exploration of cavity and curettage, which it is essential to induce osseous neoformation and in the determination of the diagnosis (12). Aggressive management must be avoided to preserve noble structures (2,16,17,22,31), mainly because the jaw noble structures like nerves are rarely damage by lesion extension, even if in the affected area, the mandibular canals are displaced (14,25,35), or the nerve lays free into the cyst (6,9). All of our fourteen patients that were treated with conservative surgery and returned to the scheduled follow up have no relapse. This same outcome was described in many series (7,16,23,25,27,32,33). Follow up time may be variable. The most of our patients obtained total healing from SBC bone defect quickly, within three months, but for some patients more time (six months or even more) was required to archive this same bone healing status. Thus, it is important for the patient to be aware of the need for follow up even after a surgical procedure.

## Conclusions

In conclusion, the SBC is an asymptomatic lesion often discovered in routine image exams in young patients. The unilocular, well defined margin with scalloped appearance is characteristic and helps the definition of diagnosis. This review suggests a different epidemiologic trend concerning to the sex, since female were more affected, and it confirms the posterior region as the more frequent location on the analyzed lesions. Finally, conservative treatment with limited exploration and curettage remains as the gold-standard treatment and should be performed with appropriate follow-up.
